# Foreign movement in one’s own body: Patients’ experiences of being awake while treated with catheter ablation—a phenomenological study

**DOI:** 10.1080/17482631.2023.2238972

**Published:** 2023-07-27

**Authors:** Ann-Katrin Nordblom, Anna Kjellsdotter, Gabriella Norberg Boysen, Mia Berglund

**Affiliations:** a Department of Cardiology, Skaraborgs Hospital Skövde, Skövde, Sweden; b Faculty of Caring Sciences, Work Life and Social Welfare, University of Borås, Borås, Sweden; c School of Health Sciences, Skövde University, Skövde, Sweden; d Research and Development Centre, Skaraborg Hospital Skövde, Skövde, Sweden; e Prehospen, Centre for Prehospital Research, University of Borås, Borås, Sweden

**Keywords:** catheter ablation, lived experience, patient perspective, phenomenology, reflective lifeworld research, supraventricular tachycardia

## Abstract

**Purpose:**

To address the consequences of living with supraventricular tachycardia and to improve the quality of treatment, there is a need to highlight patient experiences of treatment with catheter ablation. Therefore, the aim was to describe the phenomenon of catheter ablation, as it is experienced by patients being treated awake.

**Methods:**

A descriptive design was applied based on a reflective lifeworld research founded on phenomenological epistemology. Interviews were conducted between December 2021 and Mars 2022 with seven women and five men, three to twelve months after they underwent catheter ablation.

**Results:**

Patients undergoing catheter ablation while awake during treatment, which includes experiences of relying on others expertise, being actively passive, and striving to be cured. It entails experiences of having a foreign object moving in one’s body and heart and can be endured through strategies of mainly shifted one’s mental focus.

**Conclusions:**

The effort of undergoing a catheter ablation procedure is worthwhile as the confirmation of a physical curable condition that opens a future with possibilities instead of the obstacle in daily life that tachycardia entails. For the patients, an informative and caring conversation was needed that would have provided the support they lacked before and during the ablation.

For patients with episodes of supraventricular tachycardia, an electrophysiological examination is usually necessary to establish the diagnosis (Brugada et al., 2020; Page et al., 2016). Knowledge of patients’ perspectives regarding electrophysiological examinations and catheter ablation is lacking. Electrophysiological examination and catheter ablation involve a physical intervention, with the intention to localize abnormal activity, which leads to the determination of a diagnosis and decide the course of further treatment with catheter ablation during the same procedure (Brugada et al., 2020). Catheter ablation has become the first-line option, in most cases as a cure, for the two most common regular supraventricular tachycardias: atrioventricular nodal re-entry tachycardia (AVNRT) and atrioventricular re-entry tachycardia (AVRT), with success rate of 98% and 95–97%, respectively (Brugada et al., 2020). Previous studies on patients with recurring tachycardia have described an inhibited existence that involves having to give up social activities such as driving or performing leisure activities in the case of unpredictable occurrences of tachycardia (Nordblom et al., 2017; Withers et al., 2015; Wood et al., 2007). Further, these studies also described patients’ frustration of struggling to obtaining the correct diagnosis (Withers et al., 2015; Wood et al., 2007) with repeated, time-consuming visits to health care facilities and increased utility of health care resources (Carnlöf et al., 2017; Sacks et al., 2020). Overall, the literature reveals that it is problematic for patients to be referred to specialist care for assessment of their tachycardia (Carnlöf et al., 2017; Nordblom et al., 2022; Withers et al., 2015).

There are only few studies on the patient perspective from cardiac catheterization (Beckerman et al., 1995) and electrophysiological examination (Adam & Wicha, 1994), which have revealed that patients have negative experiences in this regard considering that they were performed at the time when catheter ablation was a new procedure. However, medical technology is developing rapidly and has shortened the electrophysiological examination and catheter ablation procedure without the corresponding research regarding the patient perspective. This gap in knowledge makes it important to analyse patients’ experiences of being awake during an electrophysiological examination and undergoing catheter ablation for AVNRT and AVRT to continue improving the quality of treatment and to enhance care.

According to Toombs (2001), phenomenology provides a powerful means to illuminate the experience of a body disorder—a bodily dysfunction that arises during an episode of tachycardia. Phenomenology renders explicit the dynamic relationship between the body and the world, provides insights into the disruption of space and time, and discloses the emotional dimensions of this temporary physical dysfunction (Toombs, 2001). Patients’ experiences of being awake during electrophysiological examination and treatment with catheter ablations have, to the best of our knowledge, not been described since the 1990s. Research in this context is motivated by enhancing improvements for the individual and the health care system when it comes to utilization of existing resources. The issue in focus in this paper is access to this first-line option for treatment and determining whether updated and nuanced research based on the patient’s perspective may increase knowledge and overcome barriers related to treating patients with episodes of tachycardia. Therefore, the aim of the present study was to describe the phenomenon of catheter ablation as experienced by patients who were treated while awake.

## Methods

In this study, the phenomenon catheter ablation as experienced by patients who were treated while awake was explored by utilizing the reflective lifeworld research (RLR) approach, which is based on phenomenological epistemology as described by Dahlberg et al. (2008). To establish a greater understanding of a patient’s perspective, the phenomenon was studied temporally from the time the letter for catheter ablation reaches the patient and during examination and treatment to the time spent in the ward before the procedure, during aftercare in the ward, and until discharge. According to the routines of the studied clinic, the patient is awake and regional anaesthesia is used during electrophysiological examination and catheter ablation. To access the heart’s cavity, catheters are placed in the vein from the groin and brought up to the heart. When catheters are inserted into the heart cavity and the electrophysiological examination begins, the patient is instructed to relax, rest, lie completely still, and only speak if it hurts. After treatment, the patient is required to rest in the hospital bed and is returned to the ward for checks and nursing care during continued bed rest for three hours.

The RLR approach examines the structures of lived experiences, and the phenomenon can be understood as one that is shared with other people who have had the same experience, but one that is also unique in terms of the patient’s past through memories, earlier experiences and the future perspective with their hopes and expectations (Dahlberg et al., 2008). The lifeworld, understood as the everyday world of experiences, includes both the subjective and objective worlds. It is the concrete reality that is generally taken for granted and not reflected upon in daily activities. When interviewing patients, their narratives provide the researcher an opportunity to capture their unique experiences of these patients as voices of their lifeworld (Dahlberg et al., 2008). The RLR approach enables an understanding of the phenomenon of catheter ablation as it is experienced by patients who are treated while awake, as it enables their experiences to be illuminated in depth, with an openness and sensitivity with regard to how meanings are created from a patient’s perspective.

### Open and Reflective Attitude

A descriptive design was applied, with an open and reflective attitude adopted throughout the research project (planning, organizing, and analysing data); interviews were conducted to capture patients’ experiences (Dahlberg et al., 2008). According to the RLR approach, openness implies that the researcher needs to have an open attitude and be curious to witness the phenomenon as it presents itself. Openness can be understood as a true willingness to listen to, see, and understand the phenomenon as it is experienced by the participant without any predetermined perspective. One of the RLR methods principle is bridling, which is a conscious approach by the researcher to adopt an attitude of reflection and carefulness, within effort to reduce the pace of the understanding process and stay focused on not determining what is indefinite. This entails the researchers’ understanding and pre-understanding of bridling. Bridling implies having a critical and reflective attitude towards the phenomenon of catheter ablation as experienced by patients who are treated while awake, thereby contributing to a movement towards a new understanding of the phenomenon (Dahlberg et al., 2008).

### Participants and data collection

Participants living in middle west Sweden were recruited from one specialist clinic between December 2021 and February 2022; pilot interviews were conducted in September 2020. After two pilot interviews were conducted, an initial analysis was performed to see if the questions captured the meaning of the phenomenon. This resulted in the initial question being adjusted and the direction of the phenomenon being clarified. The participants were required to have experienced an electrophysiological examination and treated with catheter ablation for AVNRT or AVRT, were aged ≥18 years, and could understand the Swedish language. Potential participants (*n* = 18) received a letter of invitation to participate, with a self-addressed stamped envelope. Seven women and five men (*n* = 12) (included in the pilot interviews), between 21 and 89 years, with experiences of episodes of tachycardia (3 months to 41 years) agreed to participate and were interviewed 3–12 months after they had a catheter ablation, with an average of 5 months. The participants selected the interview venue; four participants were conducted in one face-to-face meeting and the remaining eight were interviewed over the telephone or online. The duration of the interviews varied between 29 to 93 minutes, with an average of 63 minutes. The interviews began with the following open question: “Can you tell me about your experiences during the catheter ablation?” Thereafter, follow-up questions were posed for further exploration (e.g., *tell me more, can you give an example, how did you feel when …* , and *what do you mean by …*) and to gain deeper insights into the phenomenon. All interviews were conducted and audiotaped by the first author and transcribed verbatim, with one exception. In one interview there was a technical error with the recorder. However, the participant declined a fresh appointment as the participant has been looking forward to talk about the experiences and had waited for this moment. Therefore, the interview was continued and the first author took notes throughout the interview.

### Data analysis

The analysis was iterative and constantly moving between the whole and the parts to a new whole. The process implies going back and forth from the whole, the descriptions of the phenomenon in the transcript interviews, analysing the meanings units, and then reconstructing the whole to understand the essence of the phenomenon (Dahlberg et al., 2008). The reconstruction was characterized by the authors’ conscious approach of openness and bridling, which was implemented through reflection. To illuminate the different parts of the data, a structure of meaning units was created that responded to the aim and then these units were divided into clusters, which included meanings units that seemed to belong together and formed a further temporary pattern of meaning. Identification of clusters was an iterative process in the search for the essential meaning of the phenomenon of catheter ablation as experienced by patients who were treated while awake. In this part of the analysis, it was important not to let certain preconceptions prevail or create any notions of the phenomenon in advance. This was done by going back and forth between closeness and distance and between the parts and the whole of the interview text, with the goal of identifying something new. The essence is to understand the most abstract level of the description of the phenomenon and this can be understood as the most invariant structure of meanings associated with the phenomenon in the actual context. After the essence was identified, a description of the nuances and variances of the phenomenon was created at a more concrete level and these were contextualized with quotations from the interviews (Dahlberg et al., 2008). In the subsequent results, the essence is presented first, followed by its constitutes—that is, the meanings that constitute the actual essence.

### Ethical considerations

Permission for this study was obtained from the ethical review board (Dnr: 2019–03295). The investigation conformed to the principles outlined in the Declaration of Helsinki (World Medical Association WMA Declaration of Helsinki, 2013). Verbal and written information regarding the aim of the study was provided to all participants with the assurance of confidentiality. The interviews were voluntary, and the participants were permitted to withdraw from the study at any time. Informed consent was obtained from each participant.

## Results

### Essence of the phenomenon

Catheter ablation, as experienced by patients who are treated while awake, involves relying on another’s expertise and entails being safe and confident; on the other hand, it also entails being unprepared and vulnerable. Vulnerability includes putting oneself in the hand of others, being simultaneously objectified and confirmed. Being awake during catheter ablation implies following instructions, being actively passive, trusting and demanding to achieve simultaneously, and striving to regain health. Sensations of foreign movements in the body when the catheters are inserted into and placed in the heart, thereby leading to experiences feelings of curiosity and being terrified, endured by shifting the mental focus away from oneself. Curiosity entails the ability to distance from one’s own body in the moment, while a terrifying experience entails existential fear and anxiety. In addition, communication between staff creates both curiosity and uncertainty. It is about creating an individual story of the event, built on ideas and knowledge. The foreign environment and context instil a sense of disorientation that reinforces vulnerability. After the monitors are turned off, there is pride, gratitude, and joy at having endured the treatment and a hope for a future without the obstacles brought on by the tachycardia.

Thus, the phenomenon comprises 1) relying on expertise in a foreign environment, 2) enduring foreign movement and sensations in the body, 3) actively passive cooperation in striving to be cured, and 4) being simultaneously objectified and confirmed.

### Relying on expertise in a foreign environment

Catheter ablation, as experienced by patients who are treated while awake, means relying on expertise in a foreign environment and trusting in the knowledge and professionalism of the staff, with the expectation of being well treated well and receiving help to deal with the tachycardia. It includes striving to regain health and look at a future with possibilities instead of obstacles, which living with tachycardia may entail. To accept the offer to do a catheter ablation is experienced as putting oneself in someone else’s hands, with confidence in the knowledge of the profession; on the other hand, there is also an attitude of things leaving things up to fate. The foreign environment entails being in a hospital and listening to the technical communication between the staff in the electrophysiological lab and a noise than can be filtered as long as the atmosphere in the room indicates that everything is normal, while also wondering what the communication implies. As one participant reflected:

And so, someone in the control room said 17, yes good, and then they burned a little on 17 and so they kept on. And then they said 324 and I wondered what it was? 324, what is that? Further in or longer out or what? Up? Down? I do not need to know, but that’s what I was thinking. (P4)

To rely on expertise in this foreign environment implies trusting the expertise, despite imagining the worst. Listening to various numbers being spoken raises thoughts regarding the significance of the numbers and the patients create their own rational explanations—perhaps the numbers are coordinates to help the operator find their way inside the heart or that they correspond to the heart rate when there is a fear of cardiac arrest. One participant, with negative life-threating experience in the past, articulated fear and trust:

Then it was on 300 and then I associate it with death. But then I thought that they are five competent people here, and if that happens, then they will save me. And then I was in good hands I thought. (P8)

The communication and the activity in the room constitute a hope for a cure from the tachycardia and a future with possibilities instead of obstacles, which confirm the decision of rely on expertise.

### Enduring foreign movement and sensations in the body

Catheter ablation, as experienced by patients who are treated while awake, consists of experiences of foreign sensations and movements inside the body and the heart and enduring treatment by shifting the mental focus from oneself. Having catheters inserted into the groin results in feelings of pressure and even a sense of harsher invasiveness. As one participant reflected,

When they inserted the first instrument into my body, I thought that they would be a little careful when they send it in, but no they were not. I felt it all along my back. It became an uncomfortable feeling right there, and it lasted for only two seconds, but it was probably the only thing I thought was unpleasant. (P6)

During the electrophysiological examination, the patients experienced short, repeated palpitations, which were rather familiar but slightly different compared to their own episodes of tachycardia—a sensational, scary, and, simultaneously, exciting experience. Further, the burning sensation during the treatment is experienced differently by participants and could go unnoticed in certain patients or turn into discomfort and border on pain for short periods. The burning sensation is experiences as an itchy, warm feeling in the chest, not purely unpleasant but more like drinking a hot beverage, felt along the oesophagus. One participant described his experience in the following manner:

Though it did not hurt. What should I say? Well, kind of drinking a hot drink in the winter, stuck in the heart? A very hot drink, that’s how I can explain it. Not very nice but not very bad either. (P12)

To endure both the examination and treatment during the time as well as the physical and mental discomfort, the patients described different strategies of shifting mental focus from themselves to something else. These strategies were described as fixing their gaze and attention on a point on the ceiling or distracting themselves by listening to the interaction among the staff. This discomfort of being under treatment could cause a short-term feeling of stress or anxiety. One participant reflected on how she endured this:

There was one time that I wanted to stop the whole thing; it was very hard in the end, when they would stress my heart, and I felt in my whole body, almost as if my heart were popping out of my mouth, but it was not for a long period, and I felt that I could handle this. (P11)

Most patients related that when they felt that they have reached a limit for what they could endure, the course of treatment was over and discomfort or pain disappears.

### Actively passive cooperating in striving to be cured

Catheter ablation, as experienced by patients who are treated while awake, entails cooperation between the patient and the operator with the common goal of curing the condition. Cooperation can be understood as necessary for relieving the symptoms of the tachycardia, but also includes shortcomings. Actively passive cooperation was experienced as trusting; on the other hand, it was demanding and difficult to achieve. Consequently, there is limited opportunity for patients to ask questions, which increases the feeling of vulnerability and being left in the hands of others. One participant articulated how she experienced a (lack of) support and expressed vulnerability:

I felt completely left out, which meant that I had to concentrate on not thinking it was disgusting and… I knew they were in control; they had all the stuff and there was everything so … They did know how my heart felt, but they did not know how I felt. (P5)

Thus, as evident from the above account, being actively passive was accomplished through self-control by trusting the process and the expertise; on the other hand, it was exhausting because they were instructed to lie completely still. This was done without the support of a sedative, as they were told that sedative could cause difficulties in inducing tachycardia, which the patients saw as an impetus to endure a little bit more. Thus, active passive cooperation becomes motivating and significant when tachycardia is induced, diagnosed, and suitable for ablation. The patients’ perceptions of being actively passive included cooperation with the staff; during ablation, this was mostly mutually experienced. In the ward, the patients experienced shortcomings in cooperation if the staff did not fully do their part, by not keeping time for checks for the groin, parameters (blood pressure and pulse), removal of pressure dressings, or delivery of promised medicine.

### Being simultaneously objectified and confirmed

Catheter ablation, as experienced by patients who are treated while awake, entails the experience of being simultaneously objectified and confirmed. Being objectified and exposed in a vulnerable situation imply being unprepared. Being unprepared entails accepting an intervention without having sufficient information regarding what it involves, which causes feelings of uncertainty and a lack of support. The information a patient received prior to catheter ablation was described as neutral and fact-filled; on the other hand, it was confusing and frightening, as it was difficult to understand and relate to their own illness. Insufficient information implies unanswered questions about the unknown that is about to occur and the uncertainty of not being prepared leads to a feeling of being left out. When the letter regarding ablation was sent to patients’ who did not feel prepared, a few declined until they received the information they needed. One participant expressed his insecurity in the following manner:

I had to say no because I could not. I could not get over that feeling, and I felt very unprepared. So, I felt that I at least wanted to talk to the cardiologist, someone who explains it a little to me, what we are doing and what is to be done. (P10)

In the context of this study, being objectified implies that the heart, the centre of the body, is being handed out as an object in the hands of others. During electrophysiological examination and catheter ablation, it is possible for patients to see their own bodies on the X-ray machine and on monitors where their own hearts are imaged with the help of advanced technology. The patients express a surrealistic feeling of being objectified when they saw their own hearts visualized on the monitor; they experienced it as exciting and frightening at the same time. Being objectified entails vulnerability and exposure, which are experienced not only during preparation for ablation but also during aftercare when checking inserts in the groin, as these are performed by staff of different sexes. These checks are performed with the blanket pulled away, and patients experienced this as being exposed in a vulnerable situation, without regard to dignity, in a hospital environment where privacy is not self-evident due to dormitory rooms and unlooked doors. From being the focus of and having everyone’s attention during the ablation, the transfer to the ward takes the patient to an environment that can be described as spartan. Patients experienced that meeting with staff is good but consistently short, which leads to the perception that considering the patient perspective is lacking; there is no room for small talk and reflection; instead, the participants felt ignored. The opportunity to share the joy of being treated was not given space, and numerous questions remained unanswered. The lack of patient perspective was articulated by one participant in the following manner:

I experienced the aftercare as very spartan. I felt this patch, I thought it was a big cut at first, but it was very small … But I felt a little … abandoned, a little lonely. I wanted to share the inherent peace I experienced after the procedure to treat something that has bothered me for several years. (P10)

On the other hand, catheter ablation entails experiences of relief and a confirmation that tachycardia can be observed on monitors, that it is real, and cannot be explained by stress or anything else, a confirmation that strengthens self-esteem. After catheter ablation, joy was experienced over having undergone the treatment with all that it entailed. As one participant put it,

I felt an ego boost that if I can handle an ablation, then I can do anything. And, I mean, here I was, awake, even though I did not feel any pain. Well, when I got the anaesthesia in the groin, there was some pain, but still it was cool, you know, and when I lay there, I do not think I have ever been so in the present as now and then. (P11)

Thoughts of not experiencing tachycardia in the future are experienced with gratitude and euphoria. Although the electrophysiological examination and the catheter ablation treatment were occasionally stressful, it strengthened patients’ self-esteem as it enabled them to avoid future episodes of tachycardia and made the effort worthwhile.

## Discussion

Since studies in supraventricular tachycardia mainly focus on developing technology to optimize strategies for electrophysiological examination and catheter ablation in objective endpoints, there are gaps in the literature regarding the patient’s perspective during these procedures (Adam & Wicha, 1994; Beckerman et al., 1995; Page et al., 2016). In this study, the phenomenon of catheter ablation, as experienced by patients who were treated while awake, is explored and described to understand the patient’s perspective based on how they felt with regard to undergoing treatment that enables them to regain their health (Galvin & Todres, 2013). The main findings are characterized by reliance on expertise and being actively passive in striving to be cured. In this specific context, the expertise is dependent on the patient lying still and breathing normally; otherwise, the position of the catheters inside the heart is affected with increased risk for complications and reduced success of the treatment (Brugada et al., 2020). The result also pinpoints vulnerable situations in which the patient perspective is lacking through the experience of being or feeling unprepared, mainly expressed as need of support before the planned ablation (mostly elective procedures), but also during and after the ablation. Suffering during care is previously described (Berglund et al., 2012) when patients felt distrusted and when their perspective on illness and health was overlooked. However, knowledge is lacking in the context of being treated while awake with catheter ablation and needs to highlight shortcomings in current care to enable development. Based on this finding, it could be suggested that there is potential to improve the quality of treatment and enhance care. The medical and care perspectives make different demands on health care; improvement in care—with a focus on the patient’s perspective—is important, mainly in terms of the communication throughout the entire process from time of referral to electrophysiological examination, catheter ablation and after discharge (Bergtun et al., 2018; Liljamo et al., 2020). What is required is more explicit in relevant verbal and written information developed with a lifeworld perspective, with openness and responsiveness to the patient, including providing them the opportunity to have a dialogue with a professional with knowledge in the field—for example, a nurse. The result of this study also illuminates the feelings of relief and confirmation in being believed, pride in having managed the catheter ablation, and gratitude for a potential cure with a view to living a future without hindrance from tachycardia (Carnlöf et al., 2017; Nordblom et al., 2022; Sacks et al., 2020).

First, the identified cooperation between patient and operator highlights an important factor for successful treatment. It entails that the patient must be actively passive by striving for self-control to comply with the instructions, which were experienced with trust and a sense of security; on the other hand, it was demanding and difficult to achieve. To our knowledge, this has not been described in this context before and would likely contribute to an increased awareness of what the treatment requires of the patient and is open for the further development in terms of providing relevant support. It may be possible, in combination with dialogue, to provide some form of relaxing exercises to prepare the patient to be actively passive under the procedure. Previous research during atrial fibrillation ablation has shown promising results with non-medical interventions; that involves stimulating the patient´s own resources parallel to traditional medical treatment (pain relief)—for example, in nurse-guided relaxation and visualization responding to subjective experience (Norgaard et al., 2015). Further, intervention with virtual reality headset has shown to improve pain perception and comfort improvement (Roxburgh et al., 2021). Even though atrial fibrillation ablation differs from an ablation for AVNRT and AVRT in several aspects, these non-medical interventions could provide support for the patients during an ablation regardless of an arrhythmia.

Second, the experience of being awake under electrophysiological examination and catheter ablation was described by the participants, with initial questions and concerns about what was going to happen during the procedure, transitioned into subsequent calmness and belief in the help provided. The participants did not report painful experiences as a big issue, except from short moments. Previous research has provided an illustration of electrophysiological examinations and catheter ablation as something that is difficult for the patient to manage (Adam & Wicha, 1994; Beckerman et al., 1995). In our view, the result from the present study contributes to updated and nuanced knowledge, which may strengthen the patient’s opportunity to receive information regarding catheter ablation as an option to treatment. It is time to let patients participate in the decision to be referred to specialist care and, if suitable, further treatment with catheter ablation.

Third, this study described experiences for patients worth discussing from an existential lifeworld perspective. The focus of care is to reduce suffering, with the goal of well-being. Here, well-being is understood in a phenomenological approach as a means of being in the world as well as a felt sense of what this is like as an experience (Galvin & Todres, 2013). The theory of existential well-being opens what is important to people and, therefore, to what caring could mean, requiring a further direction for care. To experience illness when the body acts differently during episodes of tachycardia brings insecurity to the lifeworld and creates a feeling of existential homelessness, a foreign feeling to one’s own body (Gadamer, 1996). In the present study, participants expressed vulnerability, a kind of suffering, when seeking for help and being insufficient informed before and during catheter ablation. This could be considered an instrumental means of working in the care process in the studied clinic and results in a failure from the patients’ perspective; information prior to a planned catheter ablation was asked for at an earlier stage of the care process and adapted to the patient. Here, the earlier stage of the care process is understood as the first health care contact (such as primary care) prior to, and including, specialist care. Berglund et al. (2012) founded that suffering was also arise due to health care actions that neglected a holistic and patient-centred approach to care. To avoid suffering caused by health care providers, several actions can be developed and implemented, such as digital care pathway with different kind of information written as well as visible in short information films (Liljamo et al., 2020). Furthermore, the result revealed the need for information from and dialogue with someone knowledgeable in the fields before the procedure as well as after, which could be managed by nurse-managed reception. Bergtun et al. (2018) presented the need of nurse-led structured follow-up for patients who have undergone atrial fibrillation ablation, which strengthens the motive for this kind of implementation in the context of arrhythmia and ablation.

Finally, our findings suggest several developments areas for health care practitioners to improve health care. In the present study, during the hospital stay, participants experienced that the personnel had limited time at each meeting, which hampered the opportunity for them to ask questions and led to unanswered questions throughout the care period; it also limited the opportunity to share positive experiences. The restored health and process of a sort of homecoming in the participants’ own bodies was not given attention by the staff, and an increased awareness regarding this process could improve care. In our study, this result indicated that vulnerable situations included feelings of being objectified, and questions arose regarding what objectification entailed. The term has a negative connotation as something reductionist, as if the patient is dehumanized (Todres et al., 2009) in the electrophysiological lab, understood as a highly technological, specialized environment. The focus was on the heart, the object, when the subject, the patient, could watch it simultaneously. It provides focused distancing, which is necessary for certain expertise in the electrophysiological lab, such as the operator and the assistants. However, a caring nurse must protect and support the patient’s well-being in such an environment.

This leads to the next finding from this study: experiences of different kinds of strategies to endure the electrophysiological examination and catheter ablation and the associated physical and mental discomfort. The strategies mainly helped shift the patients’ mental focus from themselves to something else to help distract them and endure the procedure. According to Galvin and Todres (2013), existential dwelling is to come home to the actual situation, to hear, abide, linger, and be present with what the situations include. This could explain how certain participants described security and confidence as a mood of being peacefully grounded in the present moment, supported by a past—with episodes of tachycardias—and the openness of a future that is arriving without tachycardias. When the participants encountered being treated while awake, the extent of the past had the breadth of being genuinely curious (without previous life-threating experiences); on the other hand, they were afraid of not surviving (based on previous life-threating experiences), but still motivated with trust and hope for a positive change in their daily life. Finally, this study revealed and described the subjective and lived experiences that come to expression, which are holistic experience that entail more than physical and biological aspects.

## Methodological considerations

The terms objectivity, validity, and generalizability are assessed by considering aspects of quality and describing the scientific value of a lifeworld research study (Dahlberg et al., 2008; van Wijngaarden et al., 2017). Objectivity brings certain ethical consequences to the research, thereby avoiding skewed results, which imposes special requirements, such as the coherence criterion of validity. To conduct valid research, it is essential to follow the researcher’s reasoning throughout the study. During the entire research project, including conducting interviews and analysing data, it was important for the authors to have an open and bridled attitude (Dahlberg et al., 2008). This was made possible by reflective dialogues in the author group to be aware of one’s own preunderstanding and not to let the understanding be predetermined. The first author (AKN), a nurse in the ablation team, did not have a caring role for the participants before, during, or after the catheter ablation. During the entire process, the first author had to critically reflect and bridled her experiences as a nurse in this context and let the patient perspective be the focus in the research process. The other authors (MB, AK and GNB) have been part of the reflection. First author performed and transcribed all the interviews, strengthening the trustworthiness and consistency of the data collection. In addition, the pilot interviews enhanced the credibility of the data collection method. The interviews were conducted face-to-face in locations selected by the participants, online or over the phone. This seemed to suit everyone, considering the pandemic situation. The interview were conducted after the catheter ablation, after a minimum period of 3 months—to provide a healing period—and 12 months as the maximum to avoid any memory lapses. Conducting interviews on phone or online may have contributed to a feeling of security, thereby allowing the participants to express feelings and criticisms that might not have been expressed in a face-to-face meeting. On the other side, certain aspects may have been lost, such as noticing nonverbal signals. However, the participants expressed that the interview gave them insights that this was the missing informative and caring conversation they required before and after the catheter ablation. The interviews took an average of 63 minutes, which provided the opportunity for follow-up questions and in-depth reflection by the participants. A diverse sample of 12 participants was included and the variety among the group of patients’ who participated in the study produced rich descriptions, which enabled the authors to describe the phenomenon in its essence and constituents to deepen the overall understanding. The data analysis process was conducted initial by the first author, then by the author team. During the analysis, the authors guarded against potential bias of pre-understanding, and the rigour of the data was continuously safeguarded by constantly engaging in critical reflection discussion. Furthermore, to enhance the trustworthiness of the analysis, an iterative process created an independent structure of meaning units to meet the goal. These units were brought together into clusters that formed into a temporary pattern of meaning. The clusters were discussed by the authors and continued until sufficient reconstruction was consistent and, finally, refined the essence. Representative quotations from the participants were used to reveal connections between the interviews, analysis, and findings. In addition, the manuscript has been peer-reviewed at a seminar with other researchers, thereby contributing to the establishing the strength and validity of the results.

With regard to generalizability, the result is always contextual (Dahlberg et al., 2008). Generalizability comes from presenting the results in a structure of meaning and in quotations from the interviews (van Wijngaarden et al., 2017). The context of being treated while awake can be transferable to similar contexts, such as patients undergoing cardiac electrophysiological examination for all kinds of arrhythmia as well as cardiac catheterization during coronary angiography. Overall, the results provided meaningful information on how to better understand, as well as support and care for patients in the studied context.

### Strengths and limitations

We claim that this study is one of the first in over 20 years to examine the patient’s perspective during electrophysiological examination and treatment with catheter ablation for AVNRT and AVRT. However, further studies are recommended, as the current study has a few limitations. First, this study was conducted in a Swedish context and participants were recruited from one clinical centre. It would be interesting to include additional electrophysiological centres to address the differences and similarities on a national as well as international level, and more research in this area could provide valuable insight and synergies to existing knowledge. Second, this study has an adult population, and it would be interesting to study the experiences of younger participants’, such as teenagers, who could possibly add different perspectives to the findings of the present study. However, the age range in the present study is mainly representative of those who received catheter ablation due to AVNRT or AVRT in an electrophysiological centre.
